# The protective influence of Astragalus on rat models of preeclampsia

**DOI:** 10.1371/journal.pone.0337583

**Published:** 2025-12-12

**Authors:** Lijun Yang, Huiniu Hao, Xinhuan Zhang, Xiaorui Ren, Miao Xu, Nan Zhang, Yudan Zhang, Hailan Yang

**Affiliations:** 1 First Clinical Medical College of Shanxi Medical University, Taiyuan, Shanxi Province, China; 2 Department of Obstetrics, The First Hospital of Shanxi Medical University, Taiyuan, Shanxi Province, China; Universidad de Murcia, SPAIN

## Abstract

Preeclampsia (PE) is a high-risk hypertensive syndrome of pregnancy that occurs in the middle to late stages of pregnancy (after 20 weeks) and has become a major risk factor for maternal and fetal health and safety. Studies have shown that some components of *Astragalus* possess antioxidant and anti-apoptotic properties, which are beneficial in various diseases. The objective of this study was to investigate the effects of Astragalus on preeclampsia-like symptoms in a rat model that was induced using NG-nitro-L-arginine methyl ester (L-NAME). Pregnant rats were evaluated for blood pressure, 24-hour urinary protein excretion, the number of surviving and resorbed fetuses, placental diameter and weight, as well as fetal body length and weight. Placental growth factor (PLGF) and soluble fms-like tyrosine kinase-1 (sFlt-1) in serum were measured by enzyme-linked immunosorbent assay (ELISA). Placental and serum malondialdehyde (MDA) and nitrite, as well as serum glutathione peroxidase (GSH-Px) and superoxide dismutase (SOD) activities, were measured. The expression of mRNA B-cell lymphoma-2 (Bcl-2), Bcl-2-associated X protein (Bax), and caspase-3 were examined using real-time quantitative PCR (RT-qPCR), and proteins expression were assessed using Western blot analysis. The results showed that *Astragalus* treatment can effectively improve the symptoms and adverse pregnancy outcomes in a PE rat model. Meanwhile, it has no adverse effects on normal pregnant rats and fetuses. Furthermore, the observed effects of *Astragalus* were associated with a reduction in oxidative damage, improved vascular endothelial function, and inhibition of the Bcl-2/Bax/caspase-3 apoptosis pathway. This research indicate that *Astragalus* could serve as a promising candidate for treating PE.

## Introduction

Preeclampsia (PE), a serious pregnancy syndrome marked by high blood pressure and proteinuria, impacts approximately 2% to 8% of maternity and is a factor of maternal and neonatal illness and mortality [1]. Preeclampsia predisposes to preterm labor and fetal growth restriction, thereby elevating the infant’s susceptibility to mortality and complications [2,3]. The current pharmacological management of PE primarily focuses on the treatment of hypertension, and a specific therapeutic agent targeting the underlying pathophysiology of PE remains elusive. This lack of targeted therapy poses a substantial risk to maternal and fetal well-being. Consequently, the identification of effective medications that address the core mechanisms of PE is of paramount importance.

The precise mechanisms underlying preeclampsia remain incompletely understood. However, accumulating evidence suggests a strong association with impaired vascular endothelial function [4,5]. A characteristic feature of this disease is an increase in soluble fms-like tyrosine kinase-1 (sFlt-1) levels. sFlt-1, an antiangiogenic factor, antagonizes the activity of vascular endothelial growth factor (VEGF) and placental growth factor (PlGF), reducing their bioavailability and leading to endothelial dysfunction [6–8]. Research has shown that exogenous supplementation with nitrite (such as sodium nitrate) can partially counteract the adverse effects caused by sFlt-1. Animal studies have also demonstrated that nitrite can alleviate gestational hypertension, inhibit the abnormal elevation of sFlt-1 and VEGF, and thereby help improve vascular function [9]. Therefore, identifying drugs that protect endothelial function is crucial.

While the exact mechanisms behind PE remain uncertain, it is broadly acknowledged that dysfunction of the placenta plays a crucial role in its onset [10]. Numerous researches have indicated that apoptosis and oxidative stress are strongly associated with impaired placental function [11,12]. Oxidative stress occurs when the balance between intracellular reactive oxygen species (ROS) production and the antioxidant defense system is disrupted, which leads to cellular damage [13]. During normal pregnancy, oxidative stress markers (e.g., ROS and lipid peroxides) are maintained within a controlled range, and the antioxidant enzymes (e.g., glutathione peroxidase (GSH-Px) and superoxide dismutase (SOD) are balanced [14]. However, in PE, ROS and lipid peroxide levels are significantly elevated, while antioxidant enzyme are reduced, leading to redox imbalance and increased oxidative damage [15]. Apoptosis, a self-destructive mechanism actively initiated by cells, serves to eliminate unnecessary or damaged cells from tissues. During apoptosis, the equilibrium between the pro-apoptotic protein Bcl-2-associated X protein (Bax) and the anti-apoptotic protein B cell lymphoma-2 (Bcl-2) is crucial for determining cell fate. Under oxidative stress, Bax expression is significantly increased, whereas Bcl-2 is inhibited, leading to an imbalance in the Bax/Bcl-2 ratio and further revitalization of caspase-3, which triggers apoptosis [16,17]. Increased placental apoptosis impairs placental function, thereby compromising fetal nutrient and oxygen delivery. Disruption of the ratio of Bax and Bcl-2 and elevation of caspase-3, a key apoptotic marker, lead to excessive apoptosis in the placenta [18,19]. Therefore, the identification of interventional agents targeting the Bax/Bcl-2/caspase-3 pathway may become a new treatment for preeclampsia.

*Astragalus* is a traditional Chinese medicine containing multiple active ingredients, primarily astragaloside IV, polysaccharides, flavonoids, and saponins. These bioactive compounds exhibit diverse applications in disease management through mechanisms including anti-oxidative stress, inflammatory response inhibition, and apoptosis modulation, encompassing conditions such as diabetic nephropathy, atherosclerosis, and colorectal cancer [20–22]. Notably, astragaloside IV has demonstrated beneficial effects in cerebral ischemia-reperfusion injury, retinal iron overload toxicity, and cellular models of Parkinson’s disease by modulating the Bax/Bcl-2 ratio [23–25]. Furthermore, recent studies indicate that astragaloside IV, a key active constituent of *Astragalus*, protects preeclamptic rats by inhibiting oxidative stress and inflammatory responses [26,27]. However, the impact of *Astragalus* on preeclampsia-related symptoms and the Bax, Bcl-2, and caspase-3 in the placental of PE rats remain unclear.

To study the potential mechanism of *Astragalus* in PE, This research used the NG-nitro-L-arginine methyl ester (L-NAME)-induced PE rat model, which has been validated by Hao Huiniu et al. [28]. We evaluated the main phenotypic characteristics, oxidative stress, and angiogenesis-related factor in PE rats following *Astragalus* administration. In addition, we examined the impact of *Astragalus* on the Bax, Bcl-2, and caspase-3 in placental of PE rats. This research enhances our comprehension of how Astragalus affects PE and its possible mechanisms, thereby promoting the creation of innovative treatment approaches for managing patients with PE.

## Materials and methods

### PE model and treatments

Forty 8- to 10-week-old female Sprague-Dawley rats, were purchased from the Laboratory Animal Center of Shanxi Medical University (Shanxi, China) to establish a PE model. Under tightly controlled clean conditions, the animals have continuous access to clean water and food. The animal experimental operations involved in this research were approved by Ethics Committee of the First Hospital of Shanxi Medical University (DWLL-2025–002). After mating female rats with male rats overnight, vaginal secretions were smeared and observed under a microscope. The start of pregnancy, referred to as gestational day 0 (GD0), was determined by the detection of spermatozoa in the smears. Pregnant rats were randomly divided into four groups of 10 rats each. (1) control group: subcutaneous saline injection for 5 days from GD13 to GD17; (2) PE group: subcutaneous L-NAME (200 mg/kg) injection for 5 consecutive days from GD13; (3) PE+Astragalus group: subcutaneous L-NAME (200 mg/kg) injection for 5 days from GD13, and simultaneous oral gavage of Astragalus liquid (10 g/kg/day) for 5 days from GD15. The *Astragalus* liquid was prepared by decocting *Astragalus* tablets (Origin: Gansu Province, China; Production Batch Number: 230715004; Production License Number: Jing 20150129; Beijing Qiancao Traditional Chinese Medicine Pieces Co., Ltd.) (S4 Data.) with water and concentrating the solution to 1 g/mL, and administered by gavage at a dose of 10 g/kg/day for 5 days. (4) Astragalus group: Subcutaneous injections of physiological saline were administered for 5 days starting from GD13, along with gastric lavage of *Astragalus* solution (10 g/kg/day) from GD15 to GD19. The control group and PE group received equal volumes of physiological saline by gavage during GD15 to GD19. The *Astragalus* dose selection was referenced a prior study demonstrating that 10 g/kg/day, 20 g/kg/day, and 5 g/kg/day of *Astragalus* decoction effectively lowered blood pressure in hypertensive rats [29]. Preliminary testing in our laboratory using these doses revealed that 10 g/kg/day of *Astragalus* significantly attenuated hypertension and proteinuria in PE rats. Therefore, this dose was chosen for Follow-up experiments.

### Blood pressure and urinary protein measurement

At gestational days 0, 6, 12, 15, 18, and 20, diastolic and systolic blood pressures were measured non-invasively in rats using a rodent-specific noninvasive blood pressure meter (BW-NIBP1106, Nanjing Calvin).

At gestational days 12 and 19, beginning at 08:00 and concluding at 08:00 the following day, the rats were placed into metabolic cages for a period of 24 hours. Urine was gathered from the rats during this time. Urinary protein was measured using a total protein (TP) test kit (A045-2, Nanjing Jiancheng).

### Sample collection

On the 20th day of gestation, rats were anesthetized via inhalation using an isoflurane small animal anesthesia ventilator (HS-300S, Chengdu Taimeng). During the procedure, heart rate was monitored and oxygen was administered to ensure adequate oxygenation. Following successful anesthesia, blood samples were collected from the abdominal aorta of the mothers. Subsequently, the placenta and fetuses were extracted via cesarean section. The placentas and fetuses were then separated and processed. Next, place the placenta naturally on the operating table, ensuring it is fully spread out with the fetal side facing upward. Then, measure its diameter using a standard ruler. The placenta’s diameter and weight, along with the fetal length and weight, were recorded. Collection and storage of placental tissue. Finally, the animal was executed using cervical dislocation.

### Enzyme-linked immunosorbent assay

PlGF levels in serum were determined using the PlGF (JL11559, Jianglai Bio) enzyme-linked immunosorbent assay (ELISA) kit. Serum levels of sFlt-1 were determined using the sFlt-1 (JL48077, Jianglai Bio) ELISA kit.

### Determination of malondialdehyde, superoxide dismutase, glutathione peroxidase, and nitrite

Levels of MDA in placental tissue and serum were assessed utilizing the MDA Test Kit (A003-1, Nanjing Jiancheng). The activity of GSH-Px in serum was analyzed with the GSH-Px Assay Kit (A005-1, Nanjing Jiancheng), which involved diluting serum samples with saline at a ratio of 1:9. The assessment of serum SOD activity was conducted using the SOD Assay Kit (A001-3, Nanjing Jiancheng), with serum samples diluted with saline at a 1:4 ratio. Use the nitrite assay kit (A038-1–1, Nanjing Jiancheng) to measure nitrite levels in serum and placental tissue.

### Immunohistochemistry

After the placental tissues were embedded in paraffin, samples of 5 μm thickness were prepared using a slicer and subsequently fixed on the surface of the slides. Prior to immunohistochemical staining, the samples were first deparaffinized by xylene, followed by dehydration using a gradient ethanol solution. To enhance the interaction between antibody and antigen, EDTA solution (ZLI-9069, Zhongshan Jinqiao, Beijing, China) was used for antigen repair treatment. After this step, the samples were blocked using plasma for 20 min, and then, slides fixed with placental tissue were incubated with antibodies against Bax (60267–1-Ig, 1:400 dilution, Wuhan Sanying), Bcl-2 (68103–1-Ig, 1:1000 dilution, Wuhan Sanying), and Caspase3 (CSB-PA423088, 1:100 dilution, Chengdu Biosciences) primary antibodies were incubated at 4°C overnight. The following day, the sections were washed three times using PBS and then incubated for 30 minutes at 37°C with a species-specific secondary antibody (PV-6000, Beijing Zhongshan Jinqiao, China). DAB staining (ZLI-9019, Beijing Zhongshan Jinqiao, China) was used to observe the color development of target proteins, and then the sections were restained with hematoxylin.

### RT-qPCR analyses

Total RNA was obtained from placental tissues through a tissue homogenization method utilizing TRIzol reagent (15596026CN, Ambion), and the purity of the RNA was assessed. The RNA underwent reverse transcription to cDNA employing the Mei5bio kit (MF-166, PolymerMe). For the analysis of gene expression, real-time quantitative PCR (RT-qPCR) was conducted with the Mei5bio kit (MF787, PolymerMei) and the results were evaluated using the LightCycler system. The relative expression of the target genes were calculated using the 2^-ΔΔCT^ approach with β-actin serving as the reference gene. The sequences of the primers are detailed in Table 1.

**Table 1:** RT-qPCR primer sequence.

Gene	Primer sequence	
	Forward primer (5’_ ~_ 3’)	Reverse primer (5’_ ~_ 3’)
Bcl-2	AAACGTCCAGAGTGCTAC	CAGCCAGATTTAGGTTCA
Bax	GGCGATGAACTGGACAAC	GTGAGTGAGGCAGTGAGGA
Caspase-3	ACGAACGGACCTGTGGACCTG	AAGAGTTTCGGCTTTCCAGTCAGAC
β-actin	CACCCGCGAGTACAACCTTC	CCCATACCCACCATCACACC

### Western blot

Western blot methods and protein extraction techniques were employed to assess the expression levels of Bax, Bcl-2, and Caspase-3 in the placenta. A protein lysis buffer (AR0102 Boster) supplemented with a protease inhibitor (AR1178 Boster) was utilized to prepare the placental lysate. The protein concentration in the lysate was quantified using the BCA assay kit (AR0146 Boster). And 30 μg of protein was taken and boiled for 5 min with protein upwelling buffer (AR1112−10, Boster). Following, total protein samples were resolved using 10% and 8% sodium dodecyl sulfate-polyacrylamide gel electrophoresis, respectively. The separated proteins were subsequently transferred onto a polyvinylidene difluoride (PVDF) membrane. After a blocking step with 5% skimmed milk for 2 hours at room temperature, the membranes were incubated with antibodies against Bax (60267–1-Ig, 1:5000 dilution, Wuhan Sanying), Bcl-2 (68103–1-Ig, 1:5000 dilution, Wuhan Sanying), Caspase3 (CSB-PA423088, 1:500 dilution, Chengdu Biosciences), and β-actin (AC038, 1:10,000 dilution, Abclonal) overnight at 4°C. On the following day, the membrane underwent washing followed by a 2-hour incubation with the secondary antibody. Visualization of the target protein bands was achieved utilizing ECL chemiluminescence. For quantifying the relative expression of the target proteins againstβ-actin, gray scale analysis was conducted using Image J software.

### Data processing methods

The data collected in these experiments were quantitative, and normality tests were conducted using GraphPad Prism 9 software. The results indicated that all data followed a normal distribution. A t-test was used for comparisons between two groups, while one-way or two-way ANOVA was employed for comparisons among multiple groups. A *p*-value of less than 0.05 was considered statistically significant. All experiments were independently repeated three times to ensure the reproducibility of the results.

## Results

### The effects of Astragalus on normal pregnant rats and fetuses

**Fig 1:**
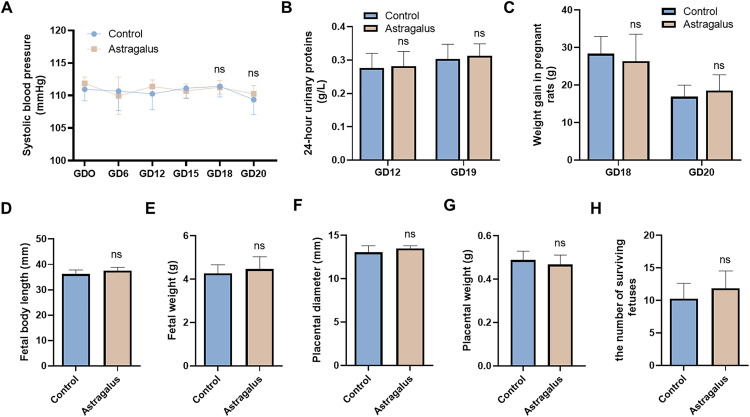
Astragalus does not have any harmful effects on normal pregnant rats or their fetuses. Non-invasive sphygmomanometry was used to measure systolic blood pressure on gestational days (GD) 0, 6, 12, 15, 18, and 20 for each group of rats **(A)**. Additionally, 24-hour urinary protein levels were measured on gestational days 12 and 19 for the respective groups **(B)**. Weight gain in pregnant rats **(C)**, fetal body length **(D)**, fetal weight **(E)**, placental diameter **(F)**, placental weight **(G)**, and the number of surviving fetuses **(H)** were measured. The study data were presented as means ± standard deviation (SD); n = 8 per group. The comparison between the two groups was conducted using t-test. *p* < 0.05 indicates a statistically significant difference. Astragalus group compared to control group;.ns represents no statistical difference.

Compared to the normal control group rats, pregnant rats treated with Astragalus showed no significant differences in systolic blood pressure (**Fig 1A**), 24-hour urinary protein (**Fig 1B**) (S5.2 Data), or weight gain on gestational days 18 and 20 (**Fig 1C**). Additionally, there were no significant differences between the Astragalus group and the control group in fetal length, fetal weight, placental diameter, placental weight, or the number of surviving fetuses (**Fig 1D–1H**). Both groups occasionally experience fetal resorption. These results suggest that Astragalus does not have any harmful effects on normal pregnant rats or their fetuses (S5.1 Data).

### Astragalus improves hypertension, proteinuria and pregnancy outcome in L-NAME-induced preeclamptic rats

**Fig 2:**
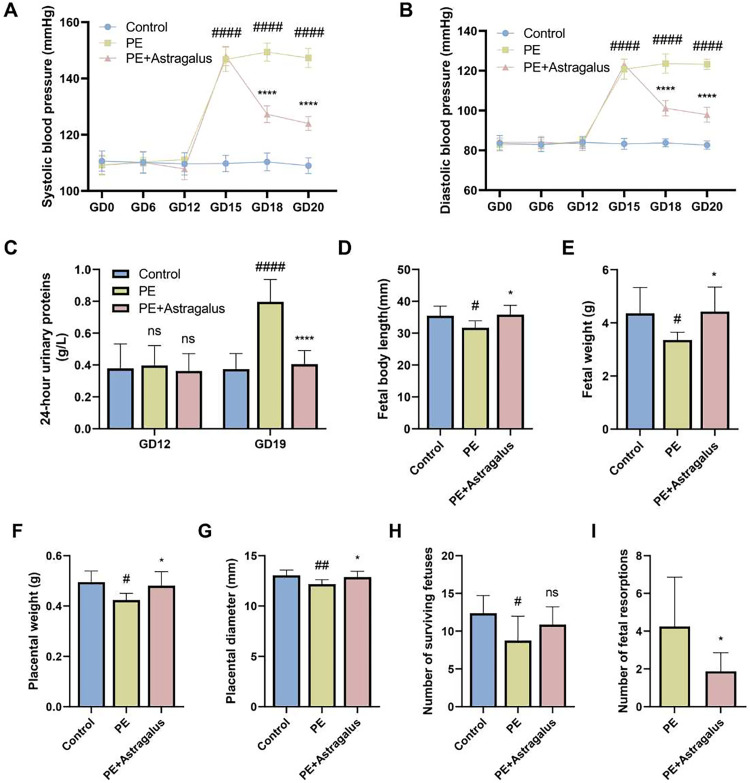
Astragalus reduces hypertension, urinary protein levels, and adverse fetal outcomes in rats with preeclampsia. Non-invasive sphygmomanometry was used to measure systolic and diastolic blood pressures on days of gestation GD0, GD6, GD12, GD15, GD18, and GD20 for each group of rats **(A and B)**. The 24-hour urinary proteins on days of gestation GD12 and GD19 for the corresponding groups were measured **(C)**. Fetal body length **(D)**, fetal weight **(E)**, placental weight **(F)**, placental diameter **(G)**, the number of surviving fetuses **(H)** and the number of fetal resorptions **(I)** were measured. The study data were presented as means ±SD; n = 8 per group. The PE group was compared with the control group; ^#^ *p* < 0.05, ^##^*p* < 0.01, ^###^ *p* < 0.001, ^####^ *p* < 0.0001, and the PE + Astragalus group compared to PE group; ^*^ *p* < 0.05, ^**^ *p* < 0.01, ^***^ *p* < 0.001, ^****^ *p* < 0.0001.

No noteworthy differences in diastolic blood pressure (DBP) or systolic blood pressure (SBP) were observed among the three rat groups at GD0, GD6 and GD12. As shown in **Fig 2A and 2B**, SBP and DBP were higher in the PE group compared to the control group at GD15, GD18 and GD20. Interestingly, *Astragalus* administration effectively reduced both DBP and SBP in preeclamptic rats at GD18 and 20 when compared to the PE group. In addition, *Astragalus* ameliorated proteinuria and improved adverse fetal outcomes in preeclampsia. The 24-hour urinary protein excretion (S6.2 Data) was comparable across the groups at GD12. However, at GD19, the PE group exhibited significantly higher 24-hour urinary protein excretion compared to the control group. *Astragalus* treatment significantly reduced urinary protein levels in the PE+Astragalus group compared to the PE group (**Fig 2C**). PE is a major risk factor for fetal health. To investigate the effect of *Astragalus* on fetal weight and length, as well as maternal placental weight and diameter, were measured. The PE group exhibited reduced fetal body length and weight compared to the control group (**Fig 2D** and 2E). Similarly, placental weight and diameter were significantly decreased in the PE group compared to the control group (**Fig 2F** and 2G). Importantly, *Astragalus* intervention restored fetal length and weight to some extent, as well as placental weight and diameter (**Fig 2D–2G**). Interestingly, we also found that, compared to the control group, the number of surviving fetuses in the PE group was significantly reduced, and the number of fetal resorptions significantly increased. However, compared to the PE group, *Astragalus* intervention did not significantly affect the number of surviving fetuses in rats with preeclampsia, but it significantly reduced the number of resorbed fetuses (**Fig 2H** and 2I). These data suggest that *Astragalus* effectively improves hypertension, proteinuria, and adverse fetal outcomes in the L-NAME-induced PE-like rat model (S6.1 Data).

### Astragalus alleviates vascular endothelial dysfunction in a PE rat model

**Fig 3:**
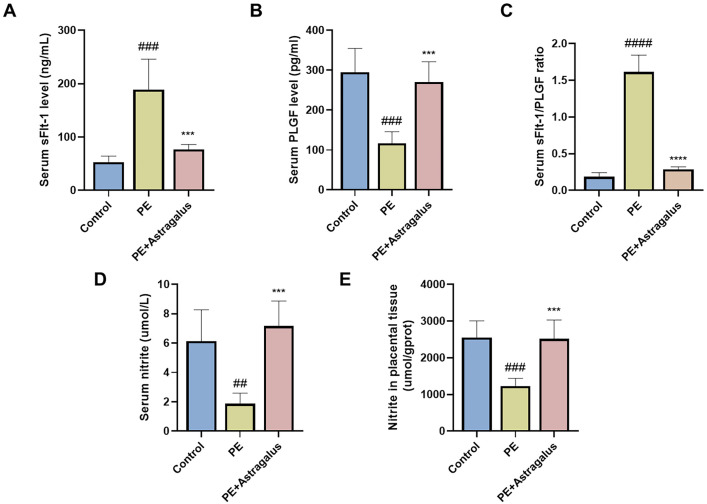
Astragalus can alleviate endothelial dysfunction associated with preeclampsia. Serum sFlt-1 **(A)** and PlGF **(B)** levels and their ratios **(C)** were measured by ELISA on gestational day (GD) 20. Use a nitrite assay kit to measure nitrite levels in serum and placental tissue **(D and E)**. The study data were presented as means ± SD; n = 5 per group. Comparison of PE group and control group; ^#^ *p *< 0.05, ^##^ *p *< 0.01, ^##*#*^ *p *< 0.001, ^####^ *p *< 0.0001. Comparison between PE+ Astragalus group and PE group;^*^ *p* < 0.05, ^**^ *p* < 0.01, ^***^ *p* < 0.001, ^****^ *p* < 0.001.

sFlt-1, PlGF, and nitrite contribute to the pathogenesis of preeclampsia by affecting vascular endothelial function. The results of this study showed increase in serum sFlt-1 concentration in the PE group compared to the control group, accompanied by a significant decrease in expression of PlGF (**Fig 3A** and 3B); further analysis of the serum samples revealed that the sFlt-1/PlGF ratio in the PE group reached nearly five times that of the control group (**Fig 3C**). However, *Astragalus* administration significantly increased serum PlGF (S7.4 Data) levels and decreased serum sFlt-1 (S7.5 Data) levels, as well as the sFlt-1 to PlGF ratio, in the PE + Astragalus group (**Fig 3A–3C**). In addition, this study demonstrated that, compared with the control group, nitrite levels (S7.2 and S7.3 Data) in the serum and placental tissue of the PE group were significantly reduced (**Fig 3D** and 3E). However, after treatment with *Astragalus*, nitrite levels increased significantly. These findings suggest that *Astragalus* treatment may substantially alleviate endothelial dysfunction caused by preeclampsia by increasing PlGF and nitrite levels while reducing sFlt-1 and the sFlt-1 to PlGF ratio (S7.1 Data).

### Effect of Astragalus on the level of oxidative stress in PE rats

**Fig 4:**
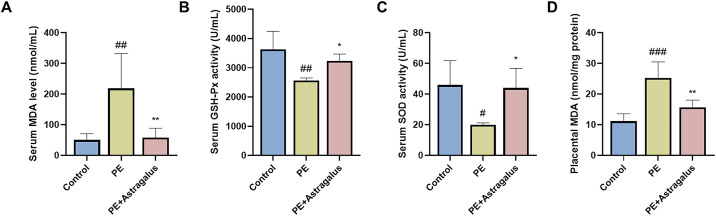
Astragalus reduces the level of oxidative stress in preeclamptic (PE) rats. Malondialdehyde (MDA; **A** and **D)** levels in serum and placenta of different groups at 20 days of gestation were determined using the corresponding kits. GSH-Px **(B)** and SOD **(C)** activities in the serum of different groups at 20 days of gestation were analyzed by using appropriate kits. The study data were presented as means ± SD; n = 5 per group. Comparison of PE group and control group; ^#^ *p* < 0.05, ^##^ *p* < 0.01. Comparison between PE+ Astragalus group and PE group; ^*^ *p* < 0.05, ^**^ *p* < 0.01.

Hypertension, placental ischemia, and hypoxia contribute to decreased antioxidant enzyme levels in PE rats, resulting in elevated oxidative damage [30]. In this study, we observed significantly increased MDA(S8.3 and S8.4 Data) levels in both the serum and placenta of PE rats (**Fig 4A** and **4D**), whereas serum GSH-Px (S8.2 Data) and SOD (S8.5 Data) were decreased (**Fig 4B** and 4C). These findings aligned with established concepts regarding oxidative stress in PE. Notably, *Astragalus* intervention significantly reduced MDA levels in the serum and placenta of PE rats (**Fig 4A** and 4D) and enhanced serum GSH-Px and SOD activities (**Fig 4B** and 4C). These results indicated that *Astragalus* intervention restored the balance between oxidative stress markers and antioxidant enzymes, thereby reducing oxidative stress levels in PE rats (S8.1 Data).

### Effects of Astragalus on Bcl-2, Bax and Caspase3 in the placenta of PE rats.

**Fig 5:**
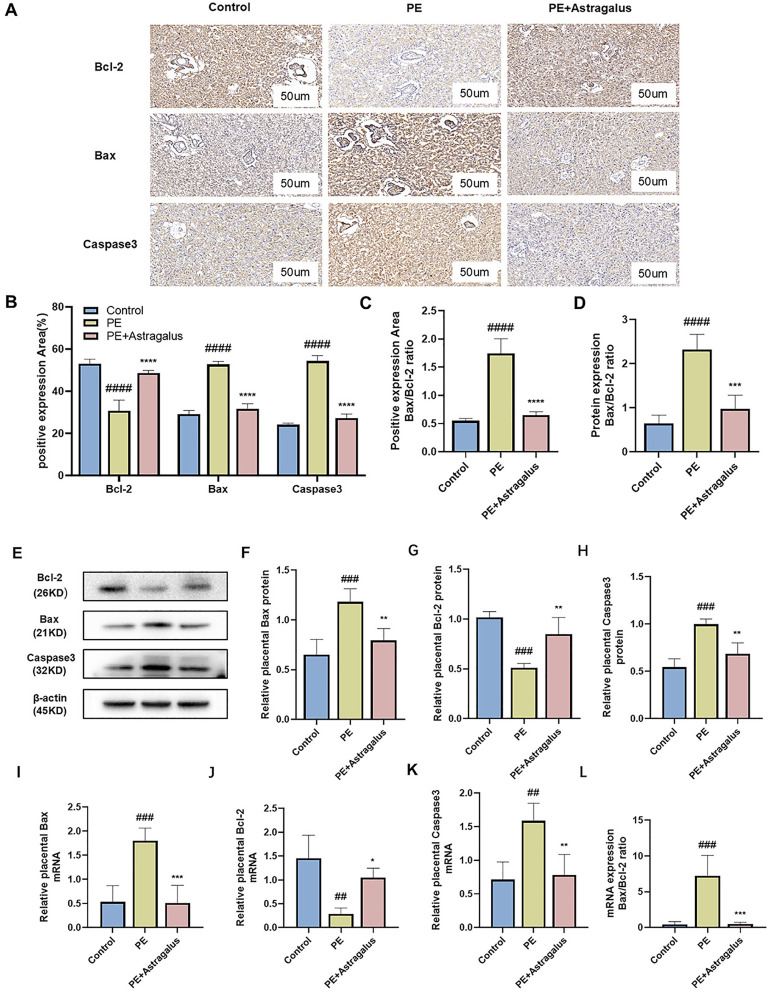
Astragalus inhibits the Bax/Bcl-2/Caspase3 pathway in the placenta of PE rats. The levels of Bax, Bcl-2, and Caspase3 in placental were analyzed by immunohistochemistry **(A and B)**, and the protein expression of Bcl-2, Bax, and Caspase3 in the placenta on day 20 of gestation was determined by Western blot **(E)**. β-actin was used as a control, and relative expression was normalized to control **(F, G** and **H)**. The mRNA expression of Bax **(I)**, Bcl-2 **(J)** and Caspase3 **(K)** in placental was detected by RT-qPCR. The Bax/Bcl-2 ratio in placental was analyzed at the tissue, protein and mRNA levels (Fig 5C, 5D, and 5L). Data are expressed as SD ± means; n = 4 per group. PE group vs. control group; ^#^ *p* < 0.05, ^##^ *p* < 0.01, ^###^*p* < 0.001, ^####^*p* < 0.0001; PE + Astragalus group compared with PE group; ^*^ *p* < 0.05, ^**^ *p* < 0.01, ^***^ *p* < 0.001, ^****^p < 0.0001.

To investigate whether the Bcl-2, Bax, and caspase-3 pathways mediated the protective effects of *Astragalus* in PE rats, we examined the tissue, mRNA, and protein expression of Bax (S1, S9.2 and S9.3 Data), Bcl-2 (S2, S9.4 and S9.5 Data), and caspase-3 (S3, S9.6 and S9.7 Data) in placental from the control, PE, and PE+Astragalus groups. As shown in Fig 5 (S9.1 Data) the placental of the PE group exhibited significantly elevated expression of Bax and caspase-3 compared to the control group, while Bcl-2 was decreased. *Astragalus* intervention significantly suppressed the expression of Bax and caspase-3 in the placental of PE rats, concurrently increasing Bcl-2 expression (Fig 5A, 5B and 5E–5K). In addition, the results demonstrated a significant increase in the Bax/Bcl-2 ratio at the tissue, protein, and mRNA levels in the PE group, whereas *Astragalus* intervention reduced this ratio (Fig 5C, 5D, and 5L). These results suggest that *Astragalus* may improve PE symptoms by regulation of Bax, Bcl-2 and caspase-3 expression in the placenta.

## Discussion

This research investigated the protective impact of *Astragalus* on PE rats and explored its mechanisms. PE is characterized by abnormally elevated proteinuria and blood pressure, often accompanied by systemic endothelial dysfunction and increased oxidative stress [31]. Oxidative stress as a central trigger for the development of preeclampsia, inducing trophoblast and endothelial cell apoptosis, leading to placental dysfunction and maternal vascular endothelial damage [32,33]. Current studies have confirmed that impaired vascular endothelial function, exacerbated oxidative stress, increased activation of Bax and caspase-3, and decreased expression of Bcl-2 are closely associated with PE development. Our study demonstrated that L-NAME successfully induced proteinuria, hypertension, and fetal growth restriction in a PE-like rat model. However, administration of *Astragalus* not only reduced proteinuria and hypertension in preeclamptic rats but also improved fetal growth restriction and decreased the number of resorbed fetuses. In conclusion, the above results suggest that the protective effects of *Astragalus* on PE may be achieved through the improvement of vascular endothelial dysfunction, reduction of oxidative stress, and amelioration of apoptosis.

The primary clinical manifestations of PE include hypertension and proteinuria. Studies have shown that placental dysplasia or dysfunction can impede the remodeling of maternal blood vessels, leading to vasoconstriction and ultimately causing blood pressure to rise [34]. In addition, PE-induced damage to renal tubules and the vascular endothelium compromises the glomerular filtration barrier, leading to proteinuria [35]. Notably, PE also significantly impacts normal fetal growth [36]. Consistent with these established PE characteristics, this study observed significantly elevated urinary protein excretion and blood pressure, as well as significantly increased fetal growth restriction, in the PE group compared to the control group. Interestingly, intervention of *Astragalus* ameliorated PE-induced proteinuria, hypertension, and fetal growth restriction. These results are similar to those that have been reported which showed that astragaloside IV, an *Astragalus* extract, improved preeclampsia symptoms and fetal pregnancy outcomes [27].

The pathophysiological mechanisms of preeclampsia involve vascular dysfunction, placental ischemia, and abnormal expression of various biomarkers, among which changes in the levels of PlGF, sFlt-1, and nitrite are particularly critical [8,37]. These factors collectively influence disease progression and clinical presentation by regulating angiogenesis and endothelial function. High levels of sFlt-1 can induce maternal vasoconstriction and placental hypoperfusion through the inhibition of VEGF and PlGF, which can lead to hypertension and other complications [38]. PlGF, a member of the VEGF family, is known to promote angiogenesis and placental development. In normal pregnancies, PlGF levels increase to support placental development and fetal growth, whereas in PE, levels are significantly decreased [39]. Nitrite is a stable oxidation product of nitric oxide (NO), and its plasma concentration reflects the generation of endogenous NO. As a key vasodilator, NO is essential for maintaining vascular homeostasis during pregnancy. In patients with preeclampsia, plasma nitrite levels are significantly reduced, indicating decreased NO bioavailability. This reduction is closely associated with pathological manifestations such as endothelial dysfunction, hypertension, and proteinuria [40]. Consistent with these research findings, the results of this study indicate that in the PE model, the levels of PlGF and nitrite significantly decreased, while sFlt-1 and the ratio of sFlt-1 to PlGF significantly increased. Interestingly, this study found that *Astragalus* intervention significantly reduced serum sFlt-1 and the ratio of sFlt-1 to PlGF, while increasing PlGF and nitrite levels. These results suggest that *Astragalus* may improve PE-induced vascular endothelial dysfunction by modulating these vascular-related factors. This finding aligns with previous research demonstrating that the primary active component of Astragalus, Astragaloside IV, reduces placental secretion of sFlt-1 and increases PlGF secretion in a PE rat model, thereby improving vascular endothelial dysfunction [26].

Oxidative stress is a pivotal factor in PE pathogenesis [41]. Excessive ROS can lead to lipid peroxidation, DNA destruction, and mitochondrial dysfunction, subsequently triggering apoptosis [42]. MDA serves as a marker of lipid peroxidation; elevated MDA levels indicate increased oxidative stress. Conversely, SOD and GSH-Px are important antioxidant enzymes, and their reduced activity leads to decreased free radical scavenging capacity, thus aggravating oxidative stress [43]. In patients with preeclampsia, MDA levels are significantly elevated, a phenomenon closely associated with oxidative stress and endothelial cell damage in placental [44]. Additionally, excessive accumulation of MDA inhibits the levels of antioxidant enzymes (e.g., GSH-Px and SOD), exacerbating oxidative stress and cellular damage [45]. Consistent with these results, the study found that MDA in serum and placental were significantly increased in preeclamptic rats, accompanied by significantly decreased GSH-Px and SOD in serum. However, *Astragalus* intervention decreased MDA in the placenta and serum of PE rats, while concurrently increasing serum SOD and GSH-Px activities, demonstrating that *Astragalus* reversed redox imbalance, enhanced antioxidant effects, and mitigated oxidative stress damage. This result is consistent with previous studies that flavonoids and polysaccharide components in *Astragalus* have been shown to have potent free radical scavenging ability and can effectively reduce cellular damage from oxidative stress [46].

To study the effect and mechanism of *Astragalus* on PE, This study examined the influence of *Astragalus* on Bcl-2, Bax, and caspase-3 in the placenta of PE rats. Apoptosis is an important pathological mechanism contributing to placental dysfunction in preeclampsia [47]. The Bax/Bcl-2/caspase-3 pathway plays a key regulatory role in apoptosis. Bax, a pro-apoptotic protein, translocates from the cytoplasm to the mitochondrial membrane upon cellular exposure to stress or injury signals. This translocation increases mitochondrial membrane permeability, facilitating cytochrome C release into the cytoplasm, which subsequently activates downstream apoptotic pathways [48,49]. Conversely, Bcl-2 protein, an apoptosis inhibitor, inhibits apoptosis by binding to pro-apoptotic proteins such as Bax, thereby preventing their activation and function [50,51]. The Bax/Bcl-2 ratio is widely regarded as an important indicator for assessing cellular apoptotic status. In preeclampsia, increased Bax expression and decreased Bcl-2 have been reported to contribute to elevated apoptosis in placental cells [52]. Caspase3 is an executor of apoptosis and its activity is significantly increased in preeclampsia [53]. Consistent with these findings, in our PE rat model, we observed upregulation of Bax and Caspase3 expression and downregulation of Bcl-2 expression in the PE rat placenta. Notably, *Astragalus* treatment dramatically upregulated Bcl-2 expression and downregulated caspase-3 and Bax in the placentas of PE rats. This suggests that *Astragalus* can improve apoptosis in PE by modulating the Bax/Bcl-2/caspase-3 pathway. This finding is in line with previous research demonstrating that *Astragalus* extracts protects neurons and attenuating apoptosis by modulating the expression of Bcl-2, Bax and caspase-3 [54].

Notably, previous studies have reported that lower doses of *Astragaloside IV* (such as 40 mg/kg and 80 mg/kg) can reduce blood pressure in PE model rats; however, this compound may exhibit maternal toxicity [26,55]. There is limited research on the efficacy and safety of *Astragalus* extract in rat models of preeclampsia. This study used a whole extract of Astragalus (10 g/kg/day) and found that it had a protective effect on PE rats. No adverse effects were observed in normal pregnant rats or their fetuses, consistent with reports that *Astragalus* injections can alleviate PE symptoms [56]. This indicates that both *Astragalus* and *Astragaloside IV* have a beneficial effect on PE. However, according to this research, the whole components of *Astragalus* are relatively safe. This may be because *Astragalus* contains multiple active ingredients, and their synergistic effects reduce the toxicity of any single component. Currently, research on the effectiveness and safety of *Astragalus* and its components in treating PE remains limited, necessitating further in-depth investigation. Additionally, research has confirmed that the pathogenesis of preeclampsia is closely related to placental cell apoptosis, but whether the whole components of *Astragalus* can improve symptoms by inhibiting this process is still a research gap. And this study confirmed its effectiveness, providing theoretical support for the treatment of preeclampsia with *Astragalus*. Finally, this study has two main limitations: (1) the components of *Astragalus* were neither isolated nor identified, leaving the specific active ingredients unknown. (2)This study utilized only animal experiments to confirm that a dosage of 10 g/kg/day of *Astragalus* is safe and effective in treating preeclampsia. However, when converting this dose to an equivalent human dose, its safety and efficacy in humans must be further validated through rigorously designed clinical trials.

## Conclusion

This study confirms the beneficial effects of *Astragalus* on PE rats, which may be attributed to the reduction of oxidative stress, improved regulation of apoptosis, and alleviation of vascular endothelial dysfunction. These research findings underscore the significant potential of *Astragalus* in ameliorating PE symptoms and enhancing maternal and neonatal pregnancy outcomes. Follow-up studies should further investigate the precise mechanisms of action and safety of *Astragalus*, as well as assess its clinical applicability, with the aim of developing novel therapeutic strategies for preeclampsia.

## Supporting information

S1 Data Bax Western blot. (PDF)

S2 Data Bcl-2 Western blot. (PDF)

S3 Data Caspase-3 Western blot. (PDF)

S4 Data Astragalus Inspection report. (PDF)

S5.1 Data Raw data. (XLSX)

S5.2 Data 24-hour urinary proteins raw data. (XLSX)

S6.1 Data Raw data. (XLSX)

S6.2 Data 24 h proteinuria raw data. (XLS)

S7.1 Data Raw data. (XLSX)

S7.2 Data Nitrite in placental tissue raw data. (XLSX)

S7.3 Data Serum nitrite raw data. (XLSX)

S7.4 Data Serum PLGF raw data. (XLSX)

S7.5 Data Serum sFlt-1 raw data. (XLSX)

S8.1 Data Raw data. (XLSX)

S8.2 Data GSH-PX raw data. (XLSX)

S8.3 Data Placental MDA. (XLSX)

S8.4 Data Serum MDA raw data. (XLSX)

S8.5 Data Serum SOD activity raw data. (XLSX)

S9.1 Data Raw data. (XLSX)

S9.2 Data Bax Western blot raw data analysis. (XLSX)

S9.3 Data Bax. PCR raw data. (XLSX)

S9.4 Data Bcl-2 PCR raw data. (XLSX)

S9.5 Data Bcl-2 Western blot raw data analysis. (XLSX)

S9.6 Data Caspase3 Western blot raw data analysis. (XLSX)

S9.7 Data Caspase3 PCR raw data. (XLSX)
