# Supplementary material for: The protective influence of Astragalus on rat models of preeclampsia
Source: PLoS One. 2025 Dec 12;20(12):e0337583. doi: 10.1371/journal.pone.0337583 (PMC12700390; doi:10.1371/journal.pone.0337583)
Supplement: S2 Data — (PDF) [file pone.0337583.s002.pdf]

Bcl-2

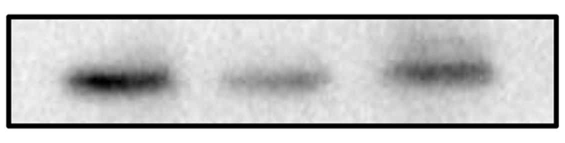

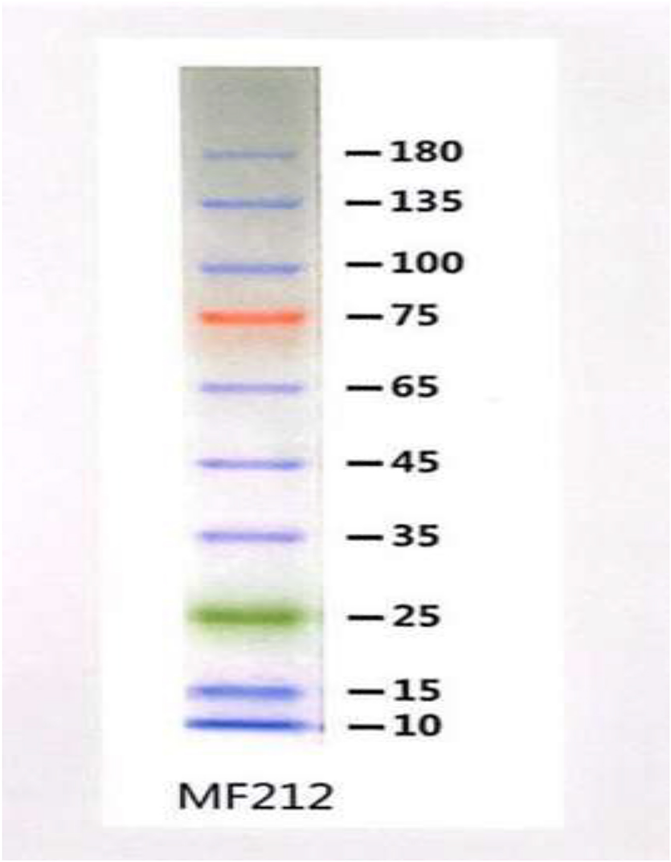

180
135
100
75
65
45
35
25
15
10
MF212

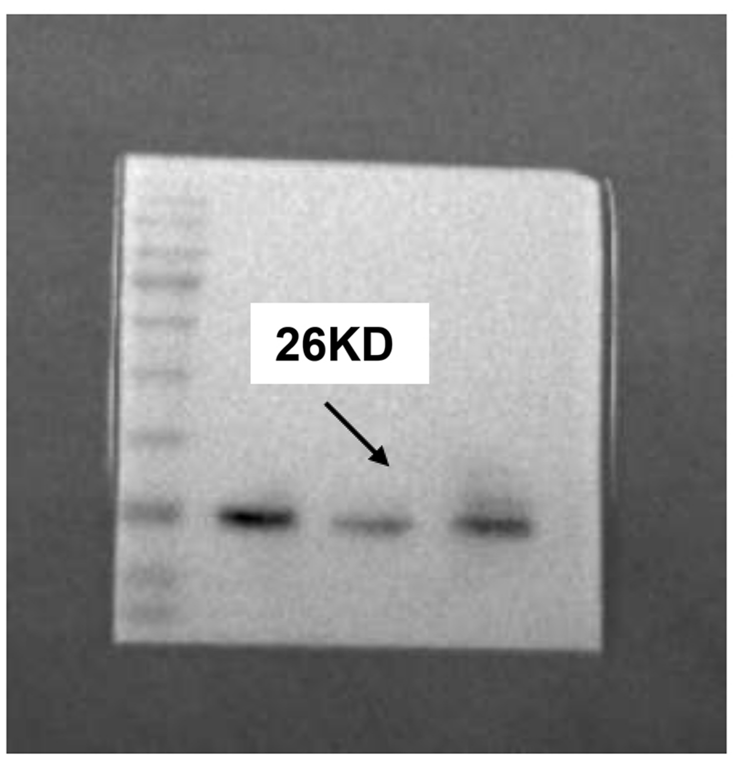
26KD

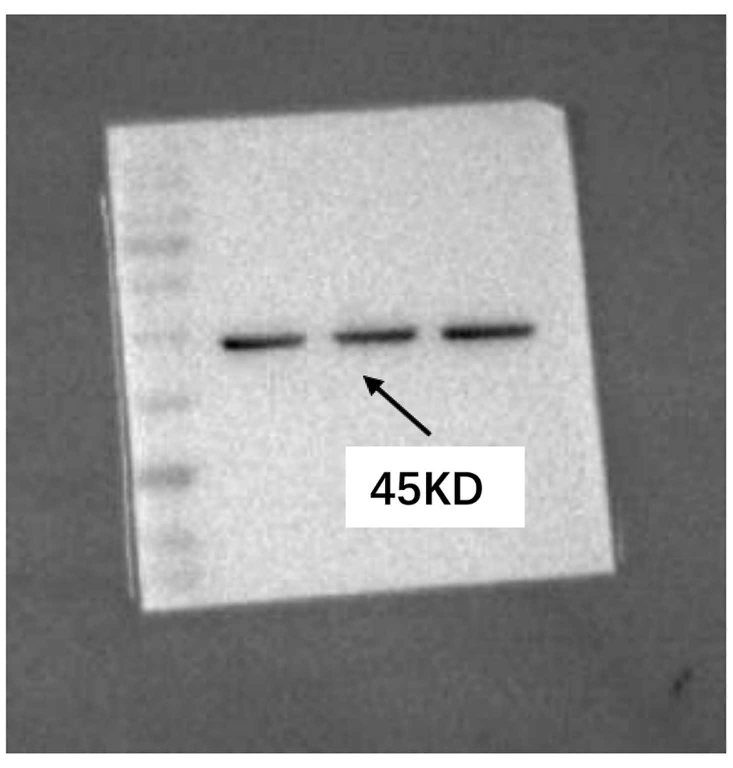
45KD

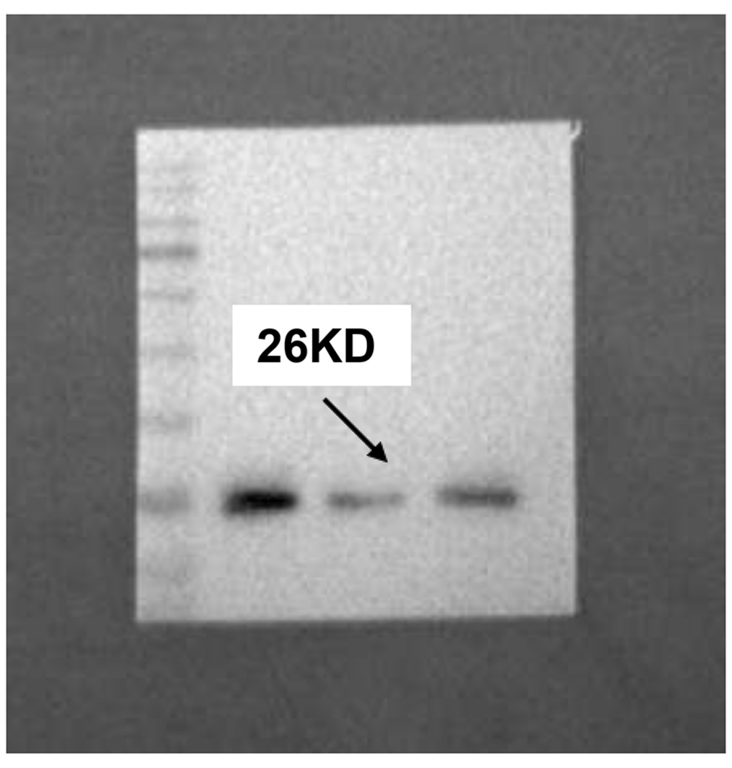
26KD

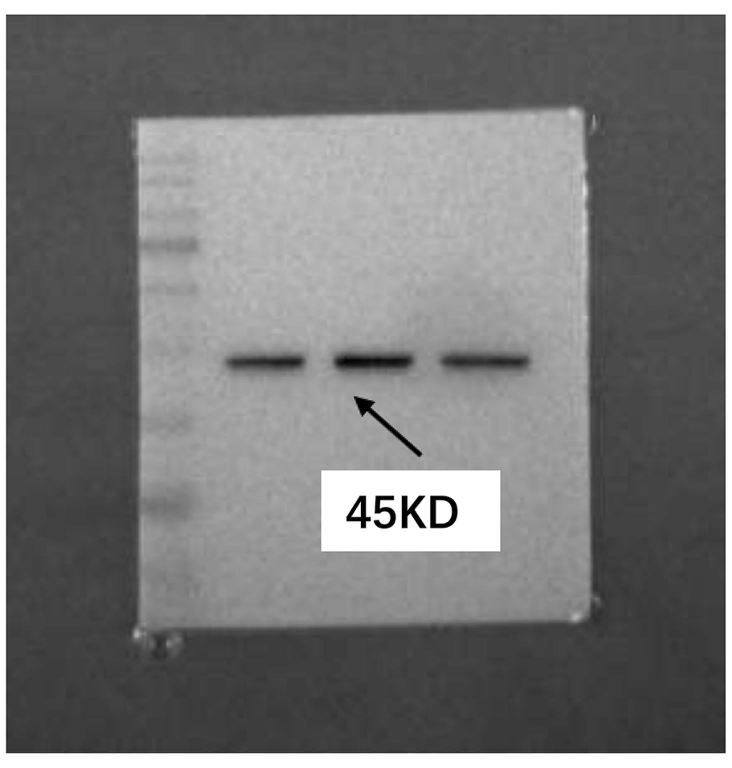
45KD

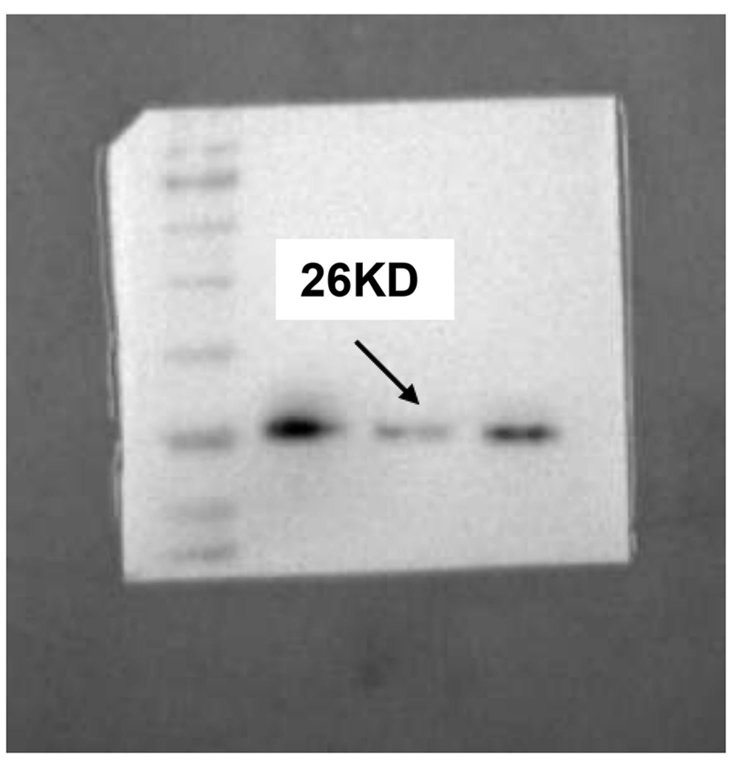
26KD

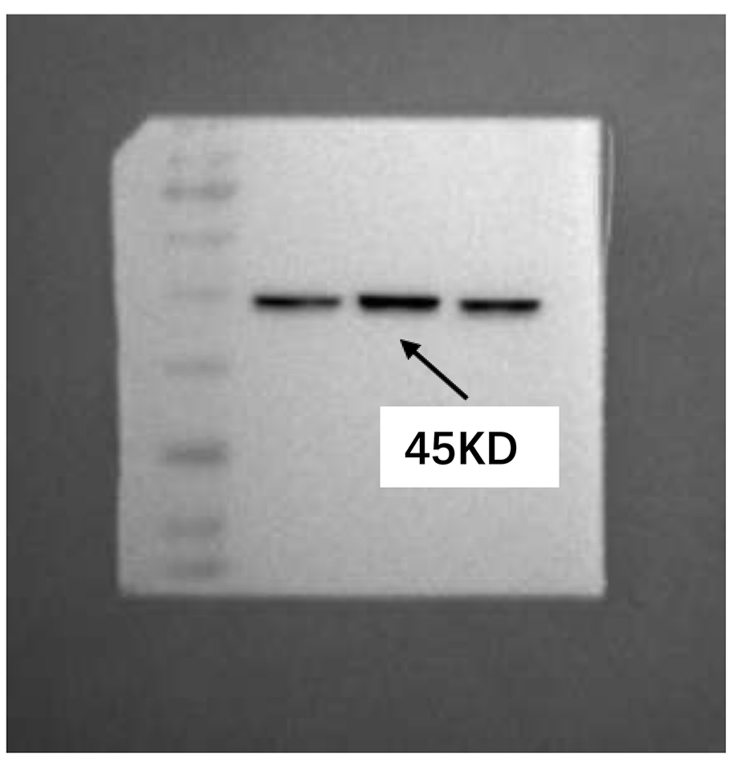
45KD

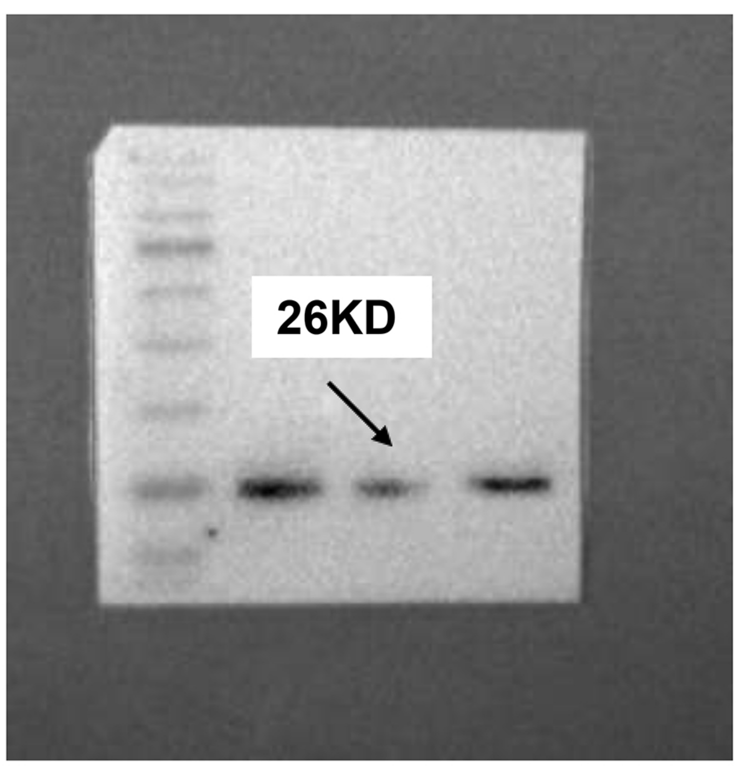
26KD

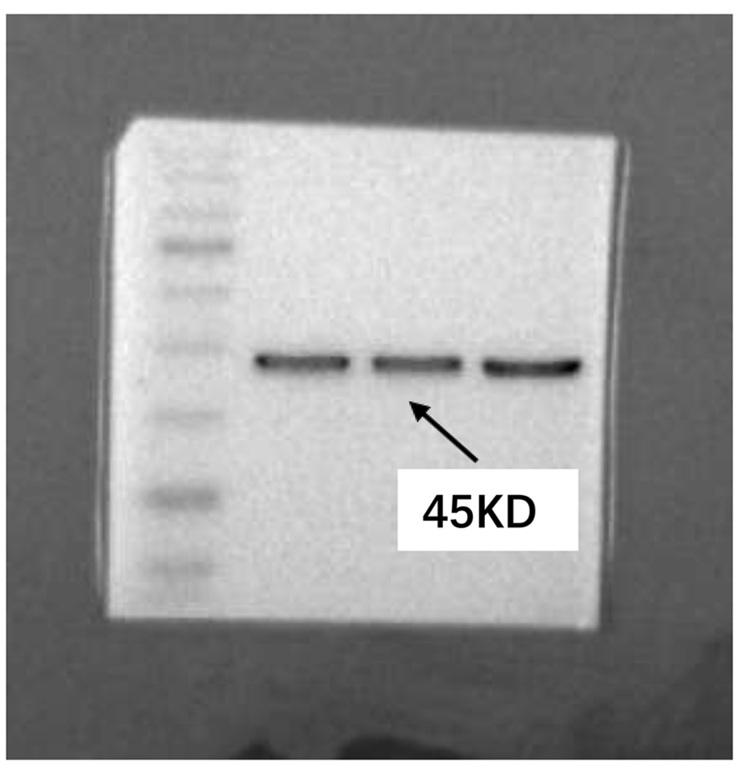
45KD

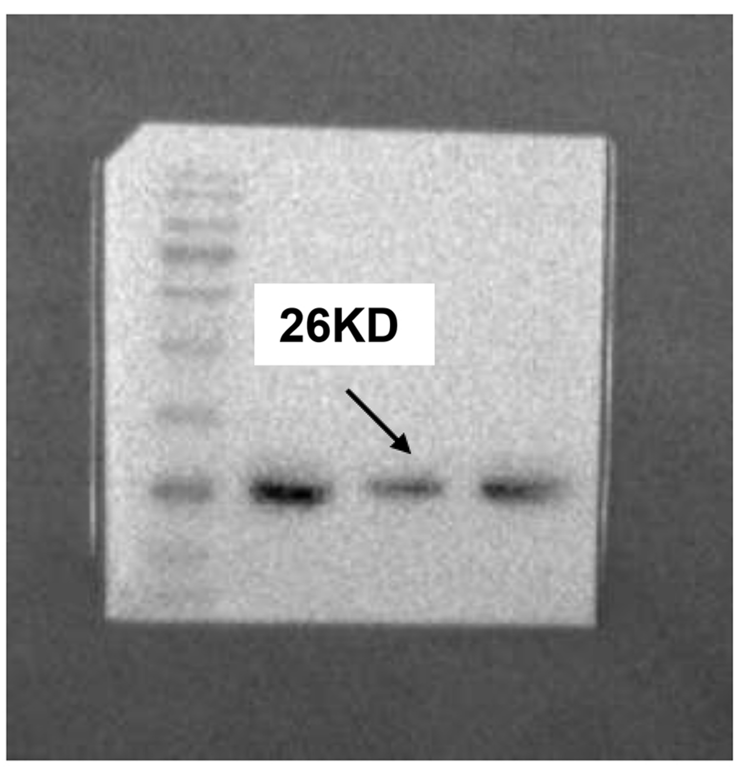
26KD

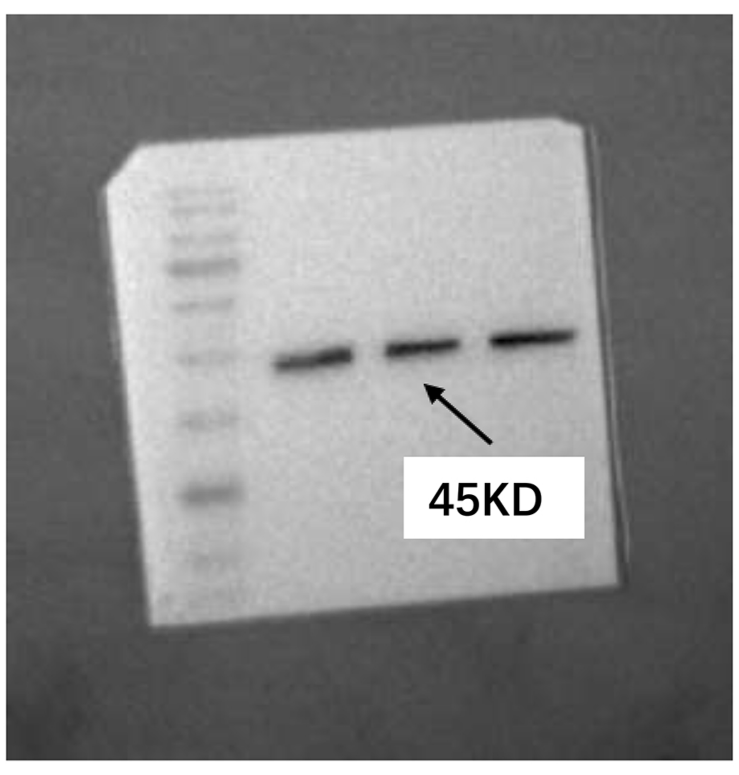
45KD

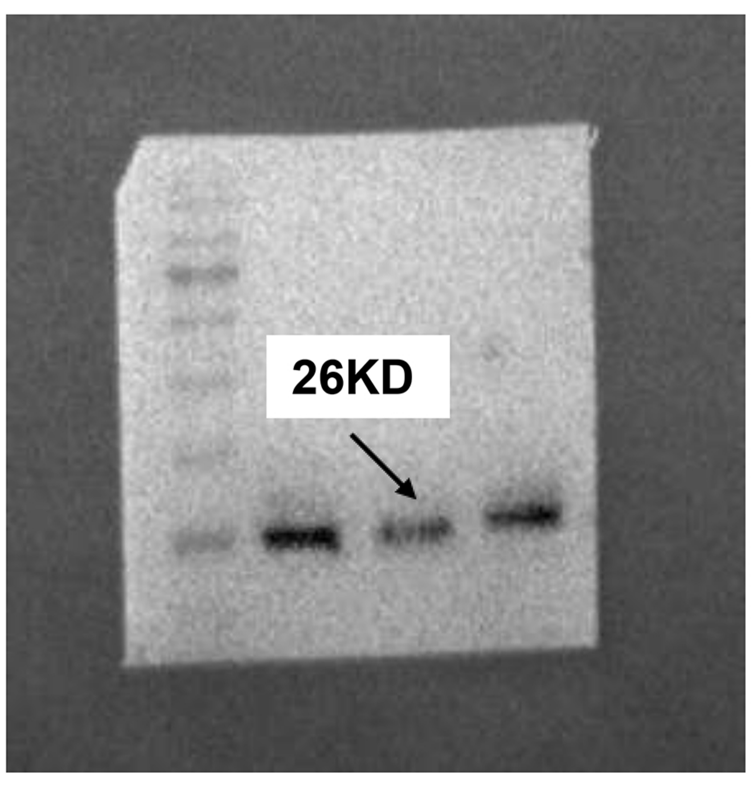
26KD

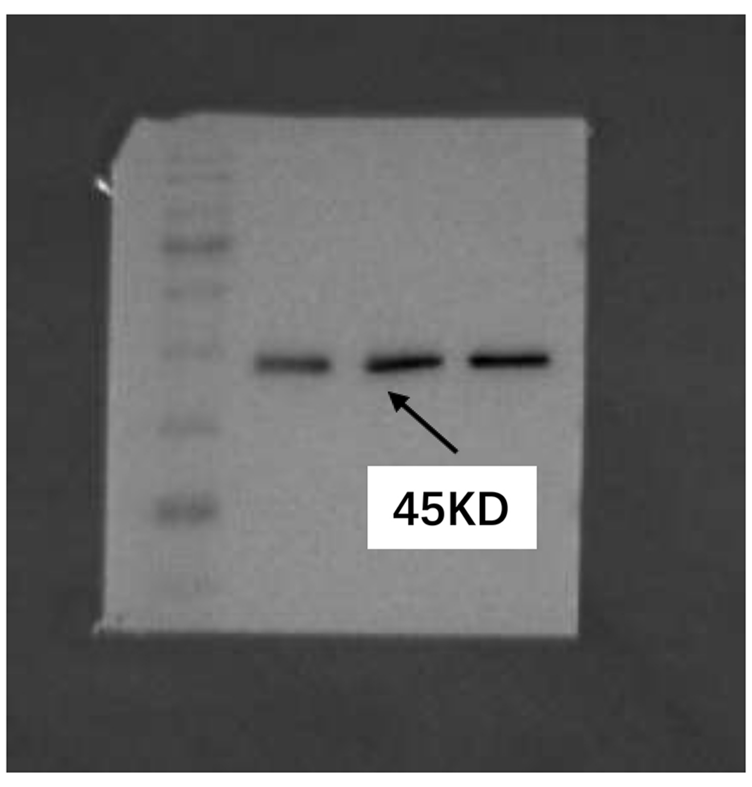
45KD

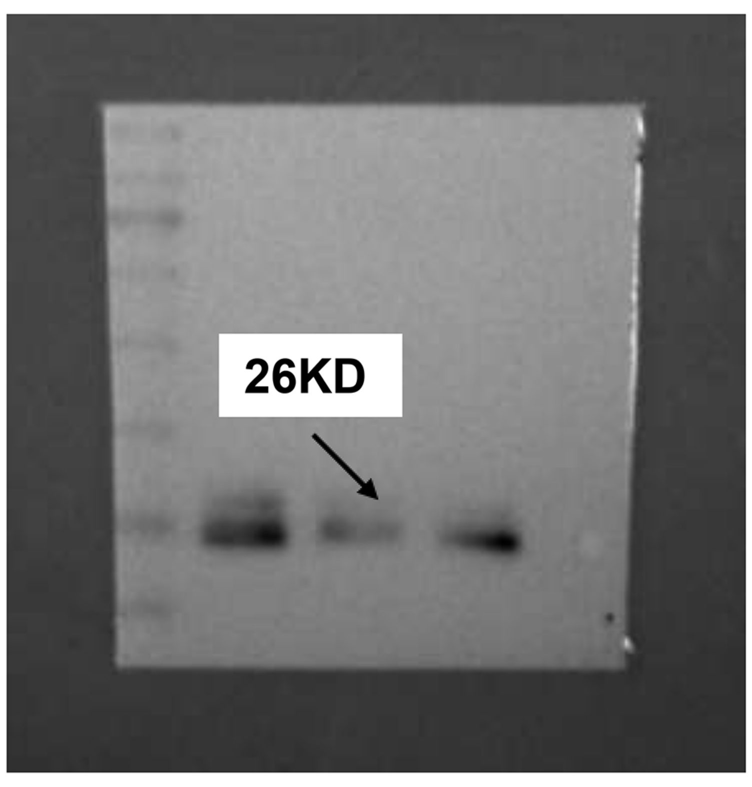
26KD

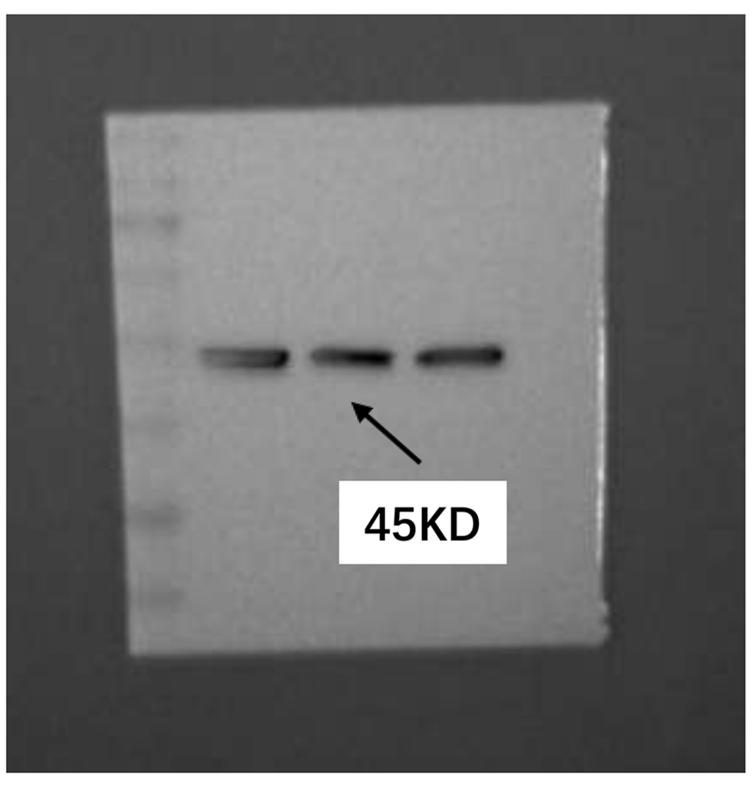
45KD
