# Supplementary material for: The protective influence of Astragalus on rat models of preeclampsia
Source: PLoS One. 2025 Dec 12;20(12):e0337583. doi: 10.1371/journal.pone.0337583 (PMC12700390; doi:10.1371/journal.pone.0337583)
Supplement: S4 Data — (PDF) [file pone.0337583.s004.pdf]

Beijing Qiancao Chinese Medicine Tablet Co.Inspection report form

Reporting Order Number: 23122902 Raw materials ☐ Auxiliary ingredients ☐ Packaging materials ☐ Finished product√

Product name	Astragalus	Batch number	230715004	Receiving number	C0101701
Manufacturing location	Gansu	Product Specification	Thick slice		
Inspection Department	Production sector	Sample size	300g		
Sampling date	September 5, 2023		Reporting date	December 29, 2023	
Detection basis	Chinese Pharmacopoeia 2020				

**Test items**	**Standardized**	**Test results**
Characterization	Corresponds to the traits of Astragalus	Corresponds to the traits of Astragalus
Distinguish	**Microscopic identification:** Should be consistent with the characteristics of Astragalus powder<br>**Thin-layer chromatography:** The color and position of the main spot of the test product should be the same as that of the control product. Astragaloside control product.<br>**Thin-layer chromatography:** The color and location of the main spot should be the same as that of the control herb of Astragalus.	Comply with regulations<br>Comply with regulations<br>Comply with regulations
Checks	Moisture content: Not to exceed 10.0%<br>Total Dust: Not to exceed 5.0%<br>Impurities: Not to exceed 3.0%<br>Heavy metals and harmful elements of lead shall: Not exceed 5mg/kg.<br>Cadmium shall not exceed 1mg/kg.<br>Arsenic shall not exceed 2mg/kg<br>Mercury shall not exceed 0.2mg/kg<br>Copper shall not exceed 20mg/kg<br>Organochlorine pesticide residues<br>Pentachloronitrobenzene (PCNB) shall not exceed 0.1mg/kg.	8.8%<br>2.5<br>0.4<br>1mg/kg<br>0mg/kg<br>0mg/kg<br>0.2mg/kg<br>12mg/kg<br>Not detected<br>Not detected
Composition determination	This product contains Astragaloside (C41H68O14) not less than 0.080%, calculated as dried product. should be less than 0.080%.<br>This product is calculated according to the dry product, containing mullein isoflavone glucoside (C22H22O10) not less than 0.020%.	0.105%<br>0.028%
Sulfur dioxide residue check	Not more than 150mg/kg	0mg/kg

Conclusion: Tested according to the Chinese Pharmacopoeia. 2020 edition, the results were in accordance with the regulations.

Inspector: Jingjing Hou Examiner: Menghan Qiao
